# Supplementary material for: In situ analysis of catalyst composition during gold catalyzed GaAs nanowire growth
Source: Nat Commun. 2019 Oct 8;10:4577. doi: 10.1038/s41467-019-12437-6 (PMC6783420; doi:10.1038/s41467-019-12437-6)
Supplement: Supplementary file 1 — Supplementary Information [file 41467_2019_12437_MOESM1_ESM.pdf]

Supplementary Information: *In situ* analysis of catalyst composition during gold catalyzed GaAs nanowire growth

Carina B. Maliakkal*[a,b], Daniel Jacobsson[a,c], Marcus Tornberg[a,b], Axel R. Persson[a,c], Jonas Johansson[a,b], Reine Wallenberg[a,c], Kimberly A. Dick[a,b,c]

[a] *NanoLund, Lund University, 22100, Lund, Sweden.*
[b] *Solid State Physics, Lund University, Box 118, 22100, Lund, Sweden.*
[c] *National Center for High Resolution Electron Microscopy and Centre for Analysis and Synthesis, Lund University, Box 124, 22100, Lund, Sweden.*

Supplementary Discussion

1. Examples of catalyst composition measured *ex situ* from Au-assisted GaAs nanowire

The catalyst composition measured post-growth depends on the conditions used to terminate the growth. Some representative examples are shown in table below.

Reference	Growth Method	Cooling / growth termination	Ga%
Jacobsson *et al.*[1]	MOCVD	Cooling in H_2 environment of zincblende nanowires (grown with relatively less Ga precursor flux)	21
		Cooling in AsH_3/H_2 environment of zincblende nanowires (grown with relatively less Ga precursor flux)	<3
		Cooling in H_2 environment of wurtzite nanowires (grown with relatively larger Ga precursor flux)	31
		Cooling in AsH_3/H_2 environment of wurtzite nanowires (grown with relatively larger Ga precursor flux)	∼3
Harmand *et al.*[2]	Molecular Beam Epitaxy	Ga stopped before As_2 after growth	1
		Ga and As_2 stopped simultaneously after growth	50
		Longer growth time to get tapered nanowires, then Ga and As_2 stopped simultaneously	31
Persson *et al.*[3]	Chemical Beam Epitaxy	Ga and As precursors stopped simultaneously after growth	9
		Cooled down in As	0
This work	MOCVD	Cooling down in AsH_3	3-4

Table 1: Ga content in the catalyst measured *ex situ* after growth of Au-seeded GaAs nanowires as reported in literature. The composition depends strongly on the growth and cooling conditions.

Typical reported values of catalyst composition measured post growth when the nanowires where cooled down in an AsH_3 environment has a relatively less spread and falls in the 0-3% range. We attempted cooling the nanowires in AsH_3 and found about 3-4% of Ga in the catalyst, tallying with earlier reports.

2. Full XEDS spectrum of Fig. 1(b) in article

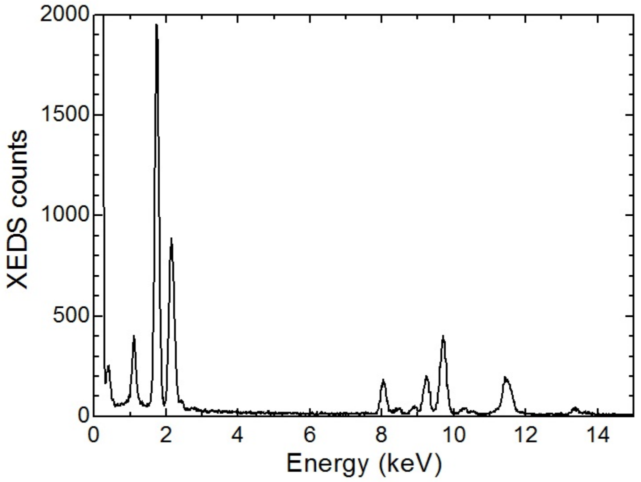

Figure 1: XEDS spectrum of the catalyst particle as shown in article Fig. 1 but with a broader x-axis range (without cropping the 3-8.2 keV range).

Fig. 1(b) in the article was cropped to show the relevant parts. A broader range of the spectrum is shown in here as Fig. 1.

3. Statistics: XEDS from different nanowires at same conditions

It is important to know that the catalyst composition across different particles is roughly the same at identical conditions. Hence we conducted experiment at 420 °C with a fixed low TMGa flow and measured the Ga concentration across different nanowire catalysts. They had roughly the same concentration. We also looked at diameter dependence of catalyst composition and found little difference in the range we measured (16 nm to 66 nm diameter during growth). (For this experiment we used Au nanoparticles with different sizes unlike the two sets of experiments reported in the article.)

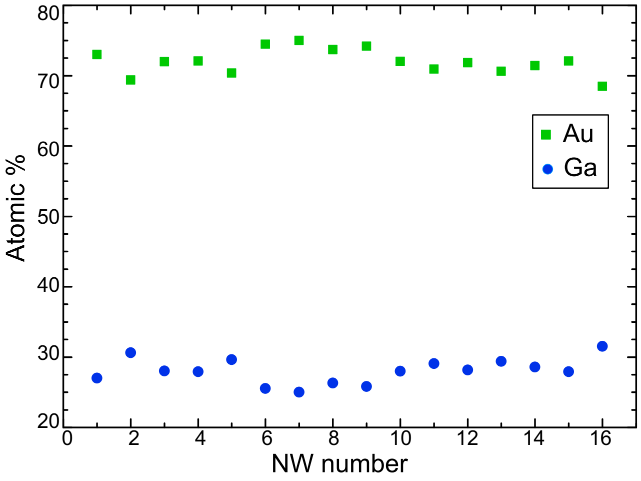

Figure 2: The Ga and Au content measured in different catalyst particles *in situ* at identical growth conditions (V/III=4600, 420 °C).

4. XEDS temperature series - II

The catalyst composition for a different nanowire as a function temperature for a fixed V/III ratio is shown below (Fig. 3). Similar to the temperature series in Fig. 2 (b) in main article, we see an increase in Ga with increasing temperature.

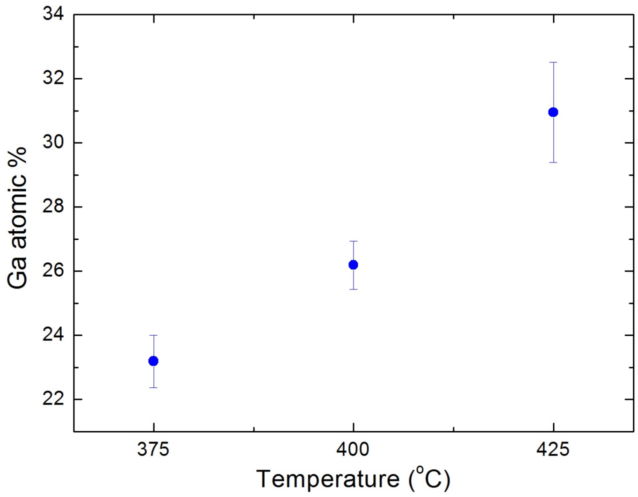

Figure 3: The Ga concentration in the catalyst of a nanowire measured as a function of temperature. Error bars show SD in the XEDS quantification.

5. Isothermal phase diagrams

Fig. 4 (a) and (b) shows the ternary Au-Ga-As isothermal phase diagrams for two temperatures – 420 °C and 500 °C. Note that the range of the plots shown here reaches 100 % for Ga and Au (x-axis), but only 0.2 % for As (y-axis). This range was chosen to clearly see the regions relevant for nanowire growth. Since for As > 0.2 % there are no new phases formed compared to the ones already seen in Fig. 4 (a) and (b), the phase diagrams can be just extrapolated linearly for that regime. According to the phase diagram in Fig. 4 (a), a single phase stable liquid alloy of Au-Ga-As exists in a very narrow range of the ternary composition near As 0.01 %, Ga 26 % and Au 74 %. At 500 °C this region is broader.

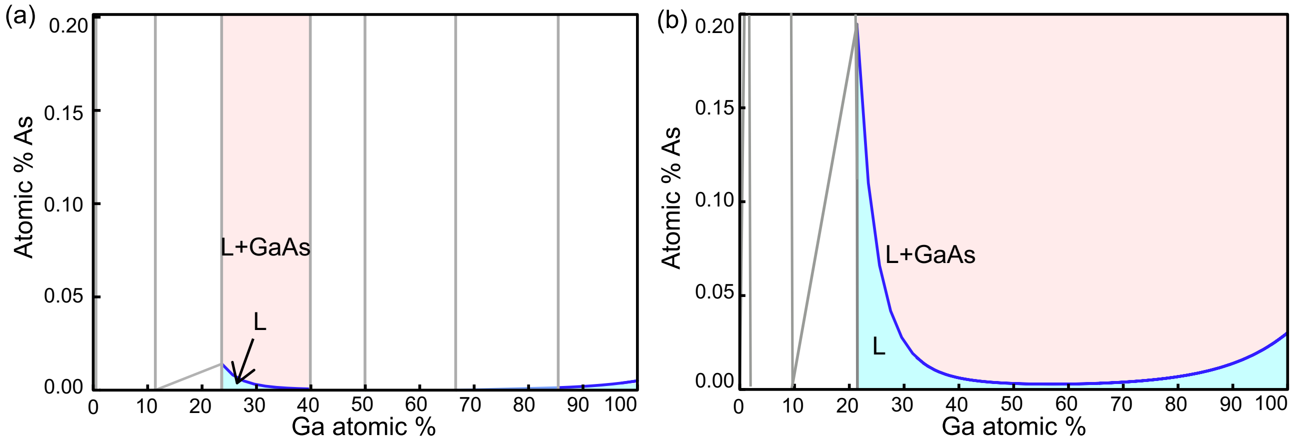

Figure 4: **Au-Ga-As ternary phase diagrams:** (a) Au-Ga-As phase diagram calculated at a fixed temperature of 420 °C. The liquidus is shown as a blue curve. The region with a stable Au-Ga-As liquid is indicated as 'L'. (b) Au-Ga-As phase diagram calculated for 500 °C

6. XEDS data measured for the temperature series and the V/III series

In the article (Fig. 2 (b) and 4 (b)) only the Ga concentration in the Au-Ga-As catalyst alloy was plotted. The XEDS quantification data measured including the background signal from the SiN_x film is shown in the tables below. Table 2 is of the temperature series shown in Fig. 2 (b) of the article. Table 3 is of the V/III series shown in Fig. 4 (b) of the article. The values in these tables are without any renormalization i.e. sum of all the observed signal, including background signal, is 100 %. (The entries in the table are in the order that the experiment was performed.) We suspect the observed weak As signal arises mainly from the adjacent GaAs nanowire. (Quantification of low energy peaks <0.5 keV are not available at 480 and 500 °C due to drastically increased strobe peak.)

T(°C)	Ga	As	Au	N	Si	Cu
440	3.5	0.2	8.0	45.1	40.3	2.9
460	5.5	0.4	11.4	25.8	52.7	4.2
480	9.2	0.7	16.8	-	67.3	6.0
500	9.6	0.8	16.5	-	67.4	5.9
420	4.6	0.6	13.5	38.8	38.0	4.4

Table 2: The XEDS quantification results for all the elements present for the temperature series measurement shown in Fig. 2 (b) of the main article.

V/III	Ga	As	Au	N	Si	Cu
4501	4.6	0.3	9.7	45.1	38.0	2.5
38349	2.5	0.4	6.3	50.5	37.9	2.3
4869	3.0	0.2	6.4	56.7	31.4	2.2
3701	3.6	0.3	8.1	52.9	32.3	2.7
2640	4.1	0.3	8.9	47.5	36.2	3.0
1769	6.2	0.5	12.1	32.6	44.4	4.2
1903	6.6	0.6	12.4	30.2	45.7	4.4
968	12.2	0.9	13.1	16.3	52.8	4.6
447	12.0	2.8	8.3	27.8	45.6	3.6

Table 3: The XEDS quantification results for all the elements present corresponding to the V/III series measurement shown in Fig. 4 (b) of the main article.

At the end of the V/III series experiment, during the XEDS at V/III=447, the nanowire changed direction and folded back onto itself. This gave rise to the relatively high As content measured at V/III=447 (Ga=52 %, Au=36 % and As=12 % with normalization Au+Ga+As= 100%) Assuming that 12 % As and hence 12 % Ga came from scattering from the GaAs nanowire, and renormalizing gives Ga=52.5 %, Au=47.5 % which is plotted in Fig. 4 (b). At the beginning of this particular XEDS scan the catalyst composition measured was Ga=51 %, Au=45 % and As=4 % (with Au+Ga+As=100 %). In similar experiments conducted there was no additional As at low V/III, and Ga% always increased at low V/III ratio. An example of another V/III series experiment is shown in the next section.

7. XEDS at 500 °C - Ga series

The catalyst composition at 500 °C as a function of the V/III ratio is shown below (Fig. 5). As expected we see an increase in Ga with decreasing V/III ratio (or increasing TMGa flow). The As content remains negligible all throughout. (This experiment was performed in the constant resistance mode of Blaze and the XEDS spectra were acquired for 2 minutes each.)

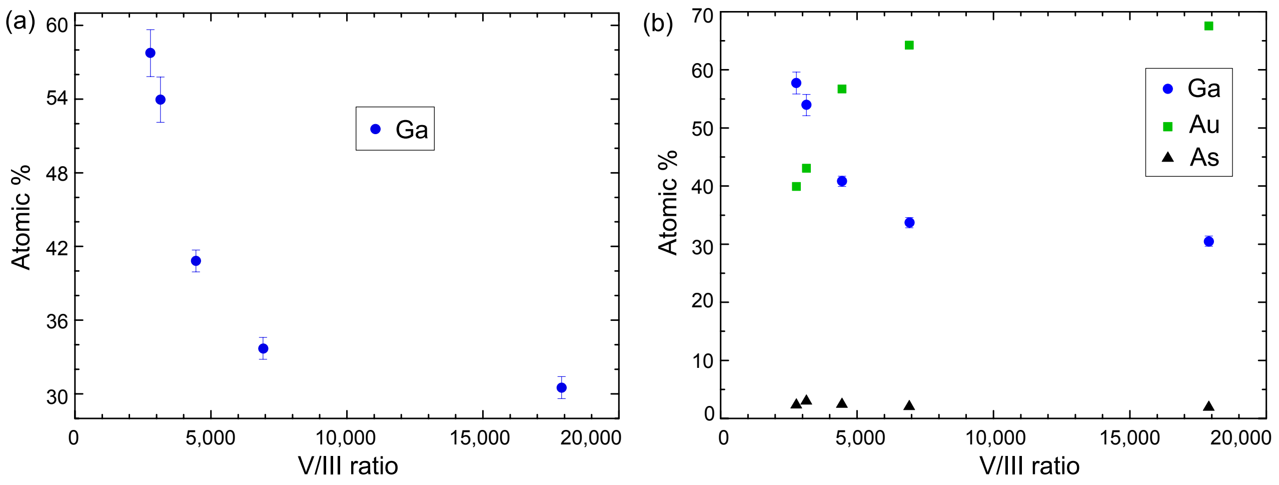

Figure 5: (a) The Ga concentration in the catalyst of a nanowire measured at 500 °C. (b) The Au and As % plotted along with the Ga % data shown in section (a). In both these plots Au+Ga+As is normalized to 100%. Error bars show SD in the XEDS quantification.

8. AsH_3 series

The Ga concentration in a catalyst where the AsH_3 flow is intentionally changed, at 420 °C, is shown in Fig. 6. (There are a few technical issues in this experiment related to actual values of the AsH_3 and TMGa partial pressures. The x-axis in this plot is the ratio of two representative lines related to AsH_3 and Ga, as measured by mass spectrometry. Qualitatively it is synonymous to the V/III ratio, but the absolute values should not be compared to other plots with V/III on the x-axis.)

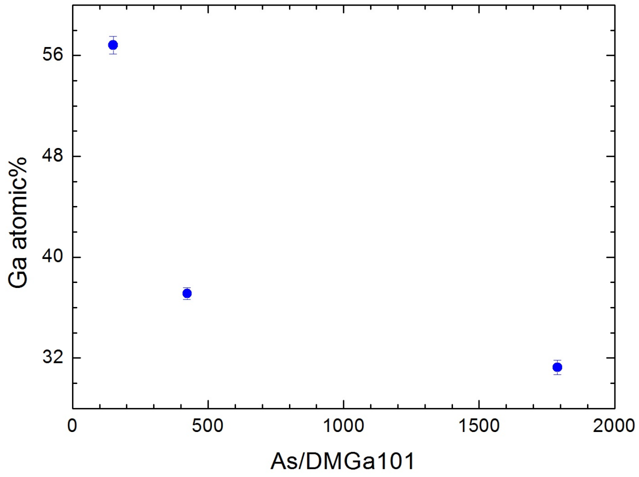

Figure 6: The Ga content of one catalyst particle measured at different AsH_3 flows. Error bars show SD in the XEDS quantification.

9. Ga from XEDS and volume change

As mentioned earlier, the volume of the catalyst particle can give an indirect estimate of the catalyst composition. This is a simple method, particularly useful if the TEM does not have any associated instruments for quantitative compositional analysis. However, this method assumes that Au is not diffusing out of the catalyst particle, which need not be true at all conditions. In cases where Au diffuses out of the catalyst, this indirect measurement becomes inaccurate. Very evident Au diffusion along Si nanowires during *in situ* experiments

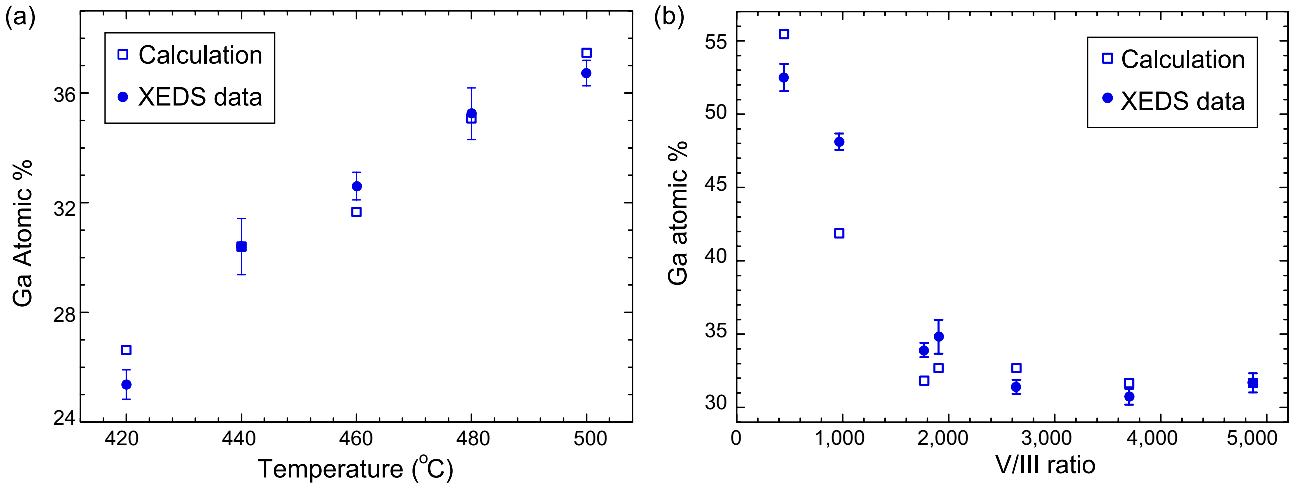

Figure 7: Estimating catalyst composition from catalyst volume change: The Ga% calculated from the measured dimensions of the catalyst particle is shown by the open squares. The Ga measured by XEDS is shown by the filled circles. (a) is for the temperature series shown in the article as Fig. 2 (b). The normalization is done with XEDS and volume measured at 440 °C. (b) is for the V/III series measured at 420 °C and shown in the main article as Fig. 4 (b). The normalization is performed at V/III=4869. Error bars in the XEDS measurement denotes SD in the quantification.

have been reported.[4] We have not noticed any obvious migration of Au in our videos, but it is in principle possible at high temperatures.[5] In some experiments (not discussed in this article) we observed tiny portions of the catalyst left on the sidewalls of the nanowire, which we do not know if it was pure Au or pure Ga or a Au-Ga mixture. Since the As concentration is very small, its contribution to the volume can be neglected. By correlating the volume change to the catalyst composition measured by XEDS, one can assess Au diffusion on GaAs nanowires at typical growth conditions.

The Ga concentration in the catalyst calculated from the volume for the temperature series experiment discussed earlier is plotted in Fig. 7 (a) as open blue squares. For this calculation we have imposed that the volume measured at 440 °C is due to 30 % Ga, which is the XEDS value measured at 440 °C. The volume was calculated by treating the catalyst particle as a spherical cap and assuming the base of the catalyst to be a circle. The measured and calculated Ga% matches, indicating that the Au diffusion is negligible. (From the catalyst volume and Ga content measured during growth, the diameter of the starting Au seed particle can be calculated to be about 28 nm, assuming the starting Au seed would have been spherical. The exact dimensions of the catalyst giving this specific nanowire that was measured is unknown to us. But the average size of the Au particles deposited on the substrate during this experiment was about 30 nm in diameter, which agrees to the 28 nm we calculated for this particular nanowire.) Similarly, Ga% calculated from the volume for the V/III series (discussed in Fig. 4 (b)) is shown in Fig. 7 (b). The normalization was done by setting the volume measured at V/III = 4869 to be due to the measured value of 31 % Ga. The catalyst shape was fitted with a prolate spheroid with the nanowire-catalyst interface being a circle.

Hypothetically, the electron beam could knock-out Ga and Au atoms from the catalyst which would cause the droplet volume to decrease steadily. However, we do not observe any obvious shrinkage of the catalyst with time at fixed growth conditions. As discussed above (by comparing the droplet size to the measured XEDS concentration), we find there is no significant loss of Au from the catalyst. In case Ga is knocked-out by the beam, the continuous supply of Ga precursor vapor will compensate for it. In short, in this study the change of catalyst composition due to interaction with the electron beam is negligible compared to the experimental error bars observed.

References

[1] D. Jacobsson, S. Lehmann, and K. A. Dick. Zincblende-to-wurtzite interface improvement by group III loading in Au-seeded GaAs nanowires. *physica status solidi (RRL) Rapid Research Letters*, 7(10):855–859, October 2013.

[2] J. C. Harmand, G. Patriarche, N. Péré-Laperne, M. N. Mérat-Combes, L. Travers, and F. Glas. Analysis of vapor-liquid-solid mechanism in Au-assisted GaAs nanowire growth. *Applied Physics Letters*, 87(20):203101, November 2005.

[3] A. I. Persson, M. W. Larsson, S. Stenström, B. J. Ohlsson, L. Samuelson, and L. R. Wallenberg. Solid-phase diffusion mechanism for GaAs nanowire growth. *Nature Materials*, 3(10):677–681, October 2004.

[4] J. B. Hannon, S. Kodambaka, F. M. Ross, and R. M. Tromp. The influence of the surface migration of gold on the growth of silicon nanowires. *Nature*, 440(7080):69, 2006.

[5] G. Otnes, M. Heurlin, M. Graczyk, J. Wallentin, D. Jacobsson, A. Berg, I. Maximov, and M. T. Borgström. Strategies to obtain pattern fidelity in nanowire growth from large-area surfaces patterned using nanoimprint lithography. *Nano Research*, 9(10):2852–2861, 2016.
